# A Policy for Addressing Menstrual Equity in Schools: A Case Study From New York City, U.S.A.

**DOI:** 10.3389/frph.2021.725805

**Published:** 2022-01-28

**Authors:** Margaret L. Schmitt, Kathleen Booth, Marni Sommer

**Affiliations:** ^1^Department of Sociomedical Sciences, Mailman School of Public Health, Columbia University, New York, NY, United States; ^2^Heilbrunn Department of Population and Family Health, Mailman School of Public Health, Columbia University, New York, NY, United States

**Keywords:** menstruation, period poverty, adolescent health, menstrual equity, menstrual health and hygiene management

## Abstract

There has been a growth in menstrual equity policy advancements in the U.S.A. in recent years; with much of the new legislation prioritizing the needs of adolescent girls in schools. New York City, a predecessor of this movement, was the first U.S.A. locality to pass such legislation in 2016. The aim of this case study was to better understand the various factors which led to the development, passage and initial implementation of New York City's *Menstrual Equity in Schools* Policy. Data collection methods included a desk review and qualitative assessment with several actors involved across the policy and introduction phases. Key findings included (1) the utility of community narratives and a pilot project as a means for overcoming initial skepticisms, proving feasibility, and generating support; (2) the importance of policy champions for overcoming fiscal objections and navigating political discourse; and (3) lessons learned from early implementation efforts, including variance in awareness and distribution models. This case study yields valuable insights into the practical considerations when designing or implementing policies aimed at tackling issues of menstrual equity within school settings.

## Introduction

Menstruation remains a highly stigmatized issue for many girls, women, and others with periods around the world. In the past decade, there has been a rapid growth in evidence on the menstruation challenges facing girls in schools from low-and-middle income countries (LMIC) (1–4). Key issues, such as insufficient access to period products, a lack of supportive and private bathrooms and inadequate menstrual health and hygiene (MHH) education, have all been found to adversely impact the health and well-being of menstruating students (1–3). Other period management challenges, such as menstrual pain, anxieties about menstrual stains and incidents of menstrual teasing can also impede girls' ability to concentrate while in class (5–8) and contribute to reduced classroom participation (9–11).

While inadequate attention has been directed toward the menstrual experiences of students in the United States of America (U.S.A.) (12, 13), a growing body of evidence has begun to document some of the challenges faced by menstruating girls and young women in schools (8, 14–16). For example, many adolescent girls, especially those from poor socioeconomic backgrounds, lack adequate menstruation education (8, 14, 17–19) and struggle to afford basic period products (15, 16, 20, 21). A survey conducted with 58 female high school students in St. Louis, Missouri found that nearly half had been unable to afford needed menstrual products at least once in the last school year (16). Further, a national survey of 471 college-attending U.S.A. women found that struggles with accessing period products were associated with experiences of depression (15).

American students that are unprepared for a menstrual period or that lack access to products while in school may have to go to a nurse or administrators' offices to obtain these items; an experience found to be daunting, embarrassing, and disruptive in terms of missing class time (22–24). A 2018 survey of 362 school nurses working in elementary, middle and high schools across the U.S.A. found that 75% of school bathrooms were not well-stocked with menstrual products (25). Unclean toilets and restrictive bathroom access policies have been cited as additional factors contributing to girls' discomfort with period management in schools (14, 25, 26). Strict bathroom policies, in particular, can result in girls being preoccupied with fears of menstrual leaks (8, 27). These anxieties are not unfounded, as incidents of students bleeding through their clothing while at school have been documented (8, 14, 17, 28).

There has been growing social and political momentum in the U.S.A. toward reframing menstruation as an issue of gender discrimination (20, 29, 30) and female empowerment (31). This includes the rise of the “menstrual equity” movement; a term used to describe the emergence of “laws and policies that ensure that menstrual products are safe and available for those who need them (29).” This movement has been magnified by advocacy demanding the repeal of period product taxes (29, 32, 33), societal calls to tackle menstrual stigma (29, 34, 35) and immediate action for addressing period poverty through free product provisions to low-income populations (20, 36). The latter has been evidenced by new city- and state-level legislation aimed at providing free menstrual products to low-income girls attending middle and high schools in several U.S.A. cities and states (20, 37).

In 2016, New York City (NYC) became the first U.S.A. locality to introduce and pass a ground-breaking package of “menstrual equity” bills (30). This pioneering bill package was comprised of separate legislative amendments for homeless services, correctional facilities and schools (38). The school-focused legislation required that all NYC Public Schools serving students in Grades 6–12 provide free menstrual products inside school bathrooms (see Box 1). Subsequently, six U.S.A. states passed similar legislation targeting students (39), including New York State in 2017 (20). Numerous other U.S.A. states have since introduced menstrual equity legislation (40).

Box 1 **Legislative text for the Menstrual Equity in Schools measure.**

Section 1. Chapter 8 of title 21-A of the administrative code of the city of New York is amended by adding a new section 21–968 to read as follows:

§ 21-968 Provision of feminine hygiene products in schools.

a. Definitions. For the purposes of this section, the following terms have the following meanings.

Feminine hygiene products. The term “feminine hygiene products” means tampons and sanitary napkins for use in connection with the menstrual cycle.

School building. The term “school building” means any facility that is leased by the department or over which the department has care, custody and control, in which there is a public school, including a charter school, serving female students in grades six through twelve.

b. The department shall make feminine hygiene products available at no cost to students in bathrooms of school buildings.

This case study examines the factors that led to the introduction and passage of the *Menstrual Equity in Schools* measure in NYC (41). As the predecessor of the growing school-focused menstrual equity legislative movements across the U.S.A., we believe that other states and municipalities may benefit from the learning generated from NYC's menstrual equity policy experience.

## Context

NYC is the largest city in the U.S.A, with a population of 8.5 million (42). NYC is one of the most diverse U.S.A cities, with 40% of its residents identifying as foreign born (43). There are high rates of poverty, with 19.5 percent of NYC residents currently living below the poverty line (44). NYC has the largest public school system in the U.S.A., serving over 1.1 million students and operated by the NYC Department of Education (DoE) (45). Politically, NYC remains a progressive environment, with the majority of city government officials identifying with the Democratic political party (46).

## Methods

We conducted a qualitative case study, including a desk review and key informant interviews with actors involved in the development and initial implementation of the school-focused menstrual equity policy. Through the two methods, we sought to identify key learning regarding the policy development process, enabling factors which supported its passage, practical learning regarding its introduction into schools, and potential implementation-related gaps or challenges requiring further attention.

### Desk Review of Media and Policy Documentation

This included a review of gray literature (media, reports) and policy-related documentation available that described the menstrual equity policy development process, legislative components, and implementation learning. Google internet searches were conducted in June through September 2019 using a variety of search terms including “menstruation,” “menstrual equity bill,” “New York City,” and “free period products in schools.” News media results were cataloged, yielding a list of 54 news articles published between 2016 and 2019.

### Key Informant Interviews (KII)

Interviews were conducted with three groups of actors (see Table 1) involved in the design, passage or implementation of the menstrual equity legislation. *Group 1*, comprised of political, advocacy and non-profit actors involved in the policy development and passage efforts; *Group 2*, included NYC DoE educators, administrators and building maintenance and operations staff currently affected by the policy and/or responsible for its implementation; and *Group 3*, involved other relevant actors including menstrual supplies manufacturers and students' parents.

**Table 1:** Study participants by role.

**Sector**	**Number of respondents**
Group 1: Government and Advocacy	6
Group 2: Education (administrative, health & logistics/operations)	10
Group 3: Other (private sector, parents)	3
Total	19

### Participant Sampling and Recruitment

All participants were recruited through purposive sampling methods (see Table 1). Group 1 participants were selected based on their involvement and knowledge of the policy across the development and legislative processes. Initial Group 1 participants were identified by the desk review and dialogue with key advocates. Recommendations were solicited from initial participants to generate additional actors. Group 2 participants (education) were purposively sampled based on (1) their position within NYC DoE Middle and High Schools (e.g., administrative, nursing, teachers) or (2) their role within relevant DoE administrative offices (Division of School Facilities, Health Education). Group 3 participants (other) were recruited based on their knowledge of or role in the passage or implementation of the policy; such actors were identified through the desk review.

### Data Collection

Two researchers conducted semi-structured qualitative interviews with 19 key informants. The research team collecting data was comprised of a co-investigator (CI) and a research assistant (RA). The CI and RA were female, with the CI having conducted previous research on menstruation and education issues. All data collection tools and methods were reviewed by the Principal Investigator (PI) prior to commencement. Interviews were conducted in-person (*n* = 2) or via phone and videoconferencing (*n* = 17) to accommodate participant preference. Data collection took place between June-October 2018. Interviews lasted ~45 min and field notes were documented by the RA. Three semi-structured interview guides were developed to capture the breadth of experience across groups. *Group One* participants were asked about their role at the time of policy development and passage, factors that led to the passage of the policy and challenges or compromises made to the policy. *Group Two* participants were asked about students' experiences managing periods at school, how the menstrual equity policy impacted school operations and implementation logistics. *Group 3* participants were asked about their involvement in the pilot project or the monitoring of the policy since introduction. The research team concluded that there was a sufficient number of key informant interviews when they detected a saturation of findings across interviews and participant group types. All participants provided oral consent. Two participants declined recording so detailed handwritten notes were generated. Participants were not compensated for their involvement. All study procedures were approved by the Columbia University Medical Center Institutional Review Board.

### Data Analysis

Two researchers (the CI and RA) reviewed the written transcripts, field notes, and desk review findings (media coverage and policy documentation) and developed a content analysis framework to analyze the interviews and identify key themes. Key themes identified were then shared with the PI for further discussion and validation. A simple code book of these key themes was then developed, and all interviews were coded using Dedoose qualitative analysis software.

## Results: Policy Phases and Elements

In exploring the passage and on-boarding of the menstrual equity policy in NYC schools, themes emerged according to three phases of the policy life cycle: (1) conceptualization and design; (2) legislative process and passage; and (3) introduction and implementation of the policy with DoE staff and students. The legislative and implementation process is provided in chronological order (see Table 2).

**Table 2:** Menstrual equity policy in NYC school timeline.

June 2015	• Community roundtables with girls, women, and community-based organization and advocates
September 2015	• Pilot project initiated in two NYC DoE High Schools in Bronx (District 9) & Queens (District 24) Boroughs
March 2016	• Free menstrual products in schools bill introduced to New York City Council. • Expansion of pilot project to include all middle and high schools in pilot districts (25 middle and high schools)
June 2016	• Public hearing held by Committee on Women's Issues on period poverty issues • Menstrual Equity bill is unanimously passed (49-0 vote)
July 2016	• Mayor Bill DeBlasio signs menstrual equity act into law
August 2016	• Implementation initiated, including installation of dispensers, training of custodial services and notifications to school leadership.

### Conceptualization and Design

#### Community Consultation

Continuous community dialogue was identified as a key component across the entire policy development process. In June 2015, before the conceptualization of the bill, NYC Councilmember Julissa Ferreras-Copeland convened a series of community roundtable discussions on menstrual health issues in low-income communities (47). This discourse sought to examine how NYC could better address issues of “period poverty” that had been increasingly highlighted by media outlets (48–50). This included exploring ideas ranging from the removal of the sales tax on menstrual products in NYC to how to alleviate the menstruation burden for low-income populations.

Community roundtables led by bill advocates provided an opportunity to capture the experiences of a diversity of NYC constituents, including groups of low-income women, students and community-based organizations across NYC. The consultations included community actors working specifically on issues of homelessness, staff from food pantries, school principals and healthcare workers. A parallel series of discussions were conducted with female DoE students. By directly gathering girls' insights, including their lived experiences with managing periods at school, the legislative team was able to better understand the potential practical impact of the policy on girls' daily lives in school. Furthermore, these discussions provided the legislative team with an opportunity to utilize compelling narratives captured directly from girls to generate buy-in with other politicians and the larger public. As one City Council staffer explained:

… *I used them [girls' insights] in every press opportunity that I got, in every talking point, and it also helped me write the actual legislation…because the girls helped me really understand. Look I haven't been in a girl's bathroom in a public school in a really long time…it helped me to really understand [the issues] …*

To further bolster the bill's eventual passage, adolescent girls and community advocates who had been part of the initial roundtables would later live testify their support for this legislation at city council hearings, and were featured in supportive media articles for the issue (51).

#### User-Informed Design

Community consultation, especially with adolescent girls, also helped to shape the bill's language and design. This included a recommended location for product distribution: student bathrooms. This specification was intentional as students had indicated that if they had a period-related emergency (e.g., an unexpected menstrual period or running out of supplies) they usually had to go to a nurse or school staff to request menstrual products. There were two advantages to shifting the distribution point to school bathrooms: (1) it removed the need for students to ask teachers or staff (gatekeepers) for a product and (2) it reduced missed class time from seeking out a product. As one non-profit advocacy actor explained: “*the rationale behind bathrooms is that it is the place where you are most likely to actually use it…it's different than putting it in a public place where you have to ask permission to access it.”* Removing school distribution gatekeepers was critical as it gave students more agency and discretion; they would not be seen asking for a product or walking back to the bathroom with it. Further, practices that relied on a school nurse for menstrual product distribution, a commonly referenced approach, were described as problematic given the oftentimes high student to nurse ratio found in NYC schools (51). Many schools were described as not having a nurse on staff every day of the school week.

#### Developing a Proof of Concept

To further build their case, bill proponents embarked on a small pilot project aimed at illustrating that the proposed policy was both feasible to implement and acceptable to students and administrators. In September 2015, a 6-month pilot project was organized on two high school campuses in two areas of NYC; one in Queens and one in the Bronx (52). HOSPECO, a private sector menstrual product and hardware manufacturer, donated product dispensers for the pilot (53). In particular, the pilot sought to examine whether directly providing free menstrual products within bathrooms was feasible given concerns about potential misuse of the products and dispensers by the student population. The pilot also sought to gain more understanding on the impact of free products on students' schooling experiences.

At the conclusion of the pilot, city council staff gathered feedback from school leadership and students through informal conversations, roundtable discussions and preliminary data collected by advocates around self-reported reductions in absenteeism. Initial findings indicated that products were both needed and respected by students, alleviating fears of misuse. One City Council actor described how the pilot alleviated concerns: “*people worried that students were going to take supplies and litter…if we could show there were kids who were mature about it and demanded it…this was the way to accomplish that.”* Beyond feasibility, the pilot also helped convince the DoE of the value of the new policy, including that it could positively impact girls' experiences in schools, such as reducing missed class time (52).

##### Persuasive Media

Beyond generating support from key community leaders and organizations, the legislative team was also eager to push this issue into the broader public consciousness. Public awareness was considered a vital strategy essential for the bill's eventual passage. This involved strategically working with advocacy groups and the local media. The legislative staff utilized the narratives collected through the community consultation processes to develop compelling messaging about the importance of its passage, using the power of the media to shape public opinion. As one City Council actor explained “*we wanted real stories, real narratives and I think if anything, that is the first driver of this.”* Between April 2015 and March 2016, a rise of media coverage was documented (50, 54–59), generating public support and awareness about the importance of addressing period poverty.

### Legislative Process and Passage

#### Legislative Champions and Rapid Introduction

Two factors were identified as critical for the successful passage of the bill: (1) the framing of menstrual products as “essential” hygiene items; and (2) the existence of a vocal champion in a budgetary leadership position. The majority of legislator opposition was related to the bill's potential budgetary impact, especially a commitment to funding menstrual products in schools over the long term. There were also concerns about the costs of set-up, including equipping bathrooms with product dispensers.

Proponents for the bill responded to these fiscal concerns by seeking to frame the issue as a basic hygiene need. This included likening period products to the provision of toilet paper, a hygiene item already included in the budget without debate. This framing of menstrual products as a necessity, as essential as toilet paper, was considered to be an effective tactic by some observers. As one City Council staffer recalled:

…*they would say things like, ‘What do you think the Board of Ed, the city schools’ toilet paper budget is? Have you thought to ask? Would you counter it? Would you say that's too much? Would you propose ways to change it? And if you've never done that before, then please don't do it on this [issue] too.'…*

Furthermore, proponents of the bill emphasized that although the upfront costs for the installation of the menstrual product dispensers would be expensive, the funding to sustain the program, namely for refilling period products, would be only a few million dollars annually (51), an amount comparable to other non-contentious hygiene expenses.

Another key influencing factor was having a key champion on the City's finance committee; in this case, the champion was the Chair. A member of the finance committee brings deep knowledge and an authority on the existing City budget. The key policy champion for the Menstrual Equity bill described the importance of her being on the finance committee, “*I came with a different kind of validity to the argument right, like nobody could tell me that we couldn't afford certain things.”* Such insights enabled the champions of the bill to respond to budgetary concerns with a high degree of knowledge and specificity. Ultimately, the bill's proponents assuaged the fiscal concerns, and the NYC City Council unanimously passed (49-0) the bill on June 21, 2016. This included a mandate to install menstrual product dispensers in 800 public schools across NYC by October 2016 (51).

### Implementation and Onboarding

#### Operationalizing Implementation

Upon passage, there were a range of activities required of the DoE to operationalize the policy. The majority of start-up and logistical tasks were the responsibility of the DoE's Division of School Facilities. This Division maintains and repairs all DoE education buildings and includes staff such as custodian engineers, handymen and cleaners. Custodial engineers played a critical role in installing menstrual product dispensers in school buildings and ensuring they were routinely refilled and properly operating over time. This includes putting in refill orders for menstrual product supplies.

School principals and school administrators were not designated as having a formal role in the implementation of the policy beyond serving as liaisons between the DoE Division of School Facilities and their own staff and students. The DoE provided email communications introducing the new menstrual product policy to school leadership, who were then responsible for disseminating it to their staff and student populations. This step was important as teachers and staff needed to know that girls no longer needed to visit a nurse or school secretary for procuring products, which could have direct implications on their school bathroom policies and student oversight. Informing students about the new policy was also critical, to assure that they were aware that products would now be available in bathrooms and free of charge. Convenient product accessibility can have direct implications on reducing students' anxiety and experiences of menstrual distress. Further, policy awareness was expected to enable students to inform school administrators and custodians about product shortages or broken dispensers.

Across these interviews conducted three years after the bill's passage, some variance was identified among school administrators and staff regarding their level of awareness of the menstrual equity policy, including some educators indicating little to no knowledge of the policy's existence. Although some school staff acknowledged this might be due to missed email correspondence by their own school's administrators, it does underscore the importance of routine reminders and information sharing about the policy.

Some concerns were also voiced regarding the lack of coordination or dialogue around the introduction of the menstrual equity policy with other relevant DoE units. This included the Health Education department which is responsible for the provision of sexual health education, including topics such as menstruation and puberty. Some Health Education staff indicated little knowledge about the new policy, suggesting that improved notice and collaboration might be beneficial. For example, it was recommended that the Health Education unit could support the creation of educational signs or flyers to accompany the free product dispensers with messaging on proper product usage and good menstrual hygiene practices, such as disposal. A few DoE school staff noted how the existing condom availability program in their schools might be an interesting model, as the program included designated spaces for distributions combined with health education on sexual health topics in high school health resource rooms (grades 9–12) (60). Staff described how these venues could be useful locations for the distribution of menstrual products and information.

#### Initial Operational Challenges

Variance was also detected in the implementation of activities. Respondents described a variety of menstrual product provision strategies being used at schools, including adaptations which moved away from the designated bathroom-based distribution plan. Some school officials indicated that while they use the new DoE issued period products, they had not altered the location of their availability from specific teachers or nurse's offices. Some teachers indicated personally purchasing products for their students from their own finances prior to the policy and welcomed the DoE products to supplement their continued classroom-based provisions. Others indicated operational challenges with placing products inside bathrooms, such as finding menstrual pads stuck to toilet stall doors, especially in bathrooms utilized by younger students.

Other implementation issues related to existing bathroom policies, many of which were shaped by broader safety and misuse issues found in school bathrooms. This included concerns around increased vaping, substance use or other misbehavior. Thus, numerous schools adopted restrictive bathroom policies which reduced students' ability to access toilets and, thus, period products. One Middle School principal described her school's strategy, explaining how “*our bathrooms are actually locked the first 10 min of class and the last 10 min of class and then throughout the day.”* Such policies can complicate a student's access to period products, which can be especially problematic in the event of an unplanned period or for students who experience heavy menstrual bleeding.

## Discussion

The NYC *Menstrual Equity in Schools* measure serves as an important example on how to respond to the menstrual needs of the community, grounding the development of a policy within the expressed concerns and requests of the population. This policy is an important building block that enables future policymakers to better address MHH in schools. This assessment also identified gaps and lessons learned, both for improving implementation across the vast NYC school system, and as learning for other localities seeking to develop and implement a similar policy.

This case study highlighted the importance of generating homegrown narratives directly from girls, women and social service providers to mobilize political will. The effectiveness of storytelling has been well-documented by other health campaigns (61–63), including within the menstrual equity arena (30, 64–67). Some menstrual equity campaigns have taken narrative advocacy a step further by adding evidence generation to highlight the scale of need among constituents. Scotland, which became the first country to mandate universal access to period products, passed their groundbreaking legislation after a 4-year grassroots advocacy campaign coupled with surveys and narratives highlighting the prevalence and experiences of period poverty (68). This compelling evidence on the scale of need bolstered support for the passage of the menstrual equity bill (69). Narrative generating strategies combined with evidence can be powerful and compelling motivators for legislative change.

Our findings indicated that the use of a small-scale pilot as “proof of concept” was an effective strategy for overcoming initial criticisms of the proposed NYC policy. This included concerns about feasibility and the potential for misuse. The use of pilots has been found effective in other contexts. Since 2016, numerous college campuses across the U.S.A. have instituted period product pilots to examine the fiscal viability of the policies and distribution approaches (70–73). The pilots have also been useful for overcoming administration concerns around potential misuse. Small-scale public product distribution pilots have also cropped up across several Canadian cities (74–77), aimed at testing new types of dispensers (78) or overcoming skepticism about potential product hoarding (79). Pilots can serve as valuable tools for not only highlighting the feasibility (fiscally and operationally) of period product policies, but also as a means for assuaging critics of proposed legislation through generating actual data and practical insights for informed decision-making.

This case study illustrated the importance of a menstrual equity champion, especially in a position of budgetary power, for the passage of this legislation. NYC is a unique context given its progressive policy environment. Thus, some of the facilitating factors and political will that motivated the passage of this bill may not be present or viable in other contexts. This may require champions in less progressive settings to rely more heavily on using community consultation, including formative research, to document the scale of need as a means for catalyzing change. Improved availability of costing data from pilots or localities currently implementing policies may also prove useful for advocates. Costing information can shed light on the fiscal implications of these policies over time and indicate the return on educational and social investment to be achieved from more comfortable and confident menstruating students.

The importance of a user-centered approach to inform policy development was another key finding of this case study. Global evidence indicates the widespread prevalence of menstrual stigma and discomfort experienced by girls in schools, especially around their need to maintain discretion (1, 3, 80). By specifying for product distribution in bathrooms, a recommendation from female students, the policy aims to enhance girls' agency in maintaining their menstrual privacy and reducing missed class time. Additional evidence from a study examining period product distributions for people experiencing homelessness further validates the importance of removing gatekeepers, as respondents indicated experiences of shame and humiliation when having to formally request for period products from service providers (81). Furthermore, product distribution strategies that depend on school nurses, common in the U.S.A, can be problematic. According to the National Association of School Nurses, only 40% of U.S.A. schools have a full-time nurse, 35% have a part-time nurse and 25% have no nurse at all (82, 83). School nursing shortages can have a detrimental impact on students' other period-related needs, such as pain management and the provision of MHH education.

The designation of bathrooms as the distribution site for free products in schools underscores the need for improved hardware options. Current bathroom product dispensers frequently break down (84–86). A 2013 survey of 1,000 American women highlighted women's reliance on public bathroom-based product dispensers in the event of an unexpected period. Nearly 50 percent of respondents indicated obtaining products from bathroom dispensers, while only 8 percent reported that the dispensers routinely worked (87). Some private sector companies are responding, as illustrated by a rise in period product dispenser innovation, such as a shift from basic coin-operated dispensers, the design norm for the past 40 years, to technology-forward machines (87).

Although not the primary focus of this case study, our findings also revealed some variance in the early implementation of the 2016 policy, including the location for product distribution. This is consistent with the 2018 findings of a Brooklyn-based Girl Scouts troop who initiated a small-scale investigation into the policy's implementation, based on their own observations of negligence in some schools. The troop surveyed public middle schools in 2 Brooklyn school districts, with only 18 percent of schools meeting the policy standards of providing menstrual products in the bathroom and disposal bins (88). They subsequently launched a formal complaint to the DoE, leading officials to vow improved oversight, including adding a new feminine hygiene protocol into existing custodial checklists (88). Public information about the current status of DoE monitoring, including the impact of the new checklists, would be useful. The monitoring challenges identified in NYC are similar to those found globally. In recent years, a number of LMIC have developed MHH policies, including for education systems. However, the measurement of progress on addressing the menstruation-related needs of female students has proven challenging (89). The Philippines provides a very useful example of a country that incorporated menstruation-related interventions into schools, similar to those in the NYC menstrual equity policy. Their policy includes reporting within the national education management information system, and a feedback loop that generates competition between schools to improve their efforts (90); the latter has served to strengthen overall implementation.

A key aspect of early implementation that emerged was the reliance on the DoE's Division of School Facilities for the management of product distribution logistics. Although custodians serve a critical role in ensuring the policy operates as intended, improved collaboration with other departments and sectors might enhance implementation efforts. This includes taking a more holistic view on addressing students' menstrual equity needs, such as related interventions around the provision of menstruation education, period pain support and access to supportive bathrooms (8, 14, 17, 80). The latter may include the availability of design measures such as discreet disposal bins, functional locks and sufficiently tall stall doors to enhance privacy, and mirrors for checking for bloodstains (91). Furthermore, our findings revealed interest in improved collaboration by Health Education actors, which might yield opportunities for social marketing of MHH education in school bathrooms alongside dispensers and disposal bins. Moving forward, menstrual equity policies could take a more expansive view of how menstruation impacts the daily lives of those in school.

## Limitations

There are a few limitations to note. First, given the narrow scope of this study, we were unable to capture the experiences and impressions of actual students, the policy's target beneficiaries. Further research is needed to capture their direct experiences with menstruation in school settings and experience with the policy. Second, the study was unable to provide extensive insights on the wide-scale implementation of the policy over time, including challenges identified, best practices and modified implementation practices. Further implementation research should examine how the policy was operationalized and the fidelity of implementation. Thirdly, recruitment proved challenging due to staff turnover and political sensitivities, however future research should continue to engage with a broad array of actors, given the useful perspectives to be gained.

## Conclusion

This case study yielded important insights regarding the facilitating factors and operational strategies utilized for the passage and early implementation of NYC's 2016 *Menstrual Equity in Schools* measure. As more cities, states and countries seek to address menstrual equity legislatively, they may benefit from learning from other menstruation policy efforts. Improved documentation from the numerous period product pilots, projects and policies being implemented around the world will enhance the capacity and success of newer initiatives. Data from the monitoring of menstrual equity policies and programs, including on best practices, challenges and the impact of such policies on student's health and well-being, may also help equip future menstrual equity advocates and policymakers. Lastly, adopting a more holistic approach to menstrual equity, one that goes beyond a focus on products to include education, menstrual pain and the role of school bathrooms would enhance the capacity of these policies to effectively address the needs of all menstruating students.

## Data Availability Statement

The raw data supporting the conclusions of this article will be made available by the authors, without undue reservation.

## Ethics Statement

The studies involving human participants were reviewed and approved by Columbia University Medical Center (CUMC) Institutional Review Board.

## Author Contributions

MSc conceived the study, supported the analysis, and drafted the manuscript. KB participated in data collection, analysis, and contributed to drafting the manuscript. MSo participated in study conception and the drafting of the manuscript. All authors read and approved the final manuscript.

## Funding

This work was supported by the Sid and Helaine Lerner MHM Faculty Fund.

## Conflict of Interest

The authors declare that the research was conducted in the absence of any commercial or financial relationships that could be construed as a potential conflict of interest.

## Publisher's Note

All claims expressed in this article are solely those of the authors and do not necessarily represent those of their affiliated organizations, or those of the publisher, the editors and the reviewers. Any product that may be evaluated in this article, or claim that may be made by its manufacturer, is not guaranteed or endorsed by the publisher.
